# Supplementary material for: The discovery of novel noncoding RNAs in 50 bacterial genomes
Source: Nucleic Acids Res. 2024 Apr 22;52(9):5152–65. doi: 10.1093/nar/gkae248 (PMC11109978; doi:10.1093/nar/gkae248)
Supplement: gkae248_Supplemental_Files [file gkae248_supplemental_files.zip › Narunsky 50 Genomes RNA Motifs Files.zip!/Publication/79-F_cylindroides/Figures/LRC-79-8-NC_021019_1_IGR_149_74357_74519.sto.pdf]

LRC-79-8-NC_021019_1_IGR_149_74357_74519.sto.r2r

5′-○○○○○○○○○○○○○○○○○○○○○○○○○○○○○A●●GAAGGCGCAAARGA●CC●CCC●●AAAAUAGY●GCUYUAUGCGUCRRCUAUUUY●C●R●CGGYRU●CU●YAU●●A●AAUR
